# Laparoscopic cholecystectomy using a wireless intelligent endoscopic system versus a conventional wired system: a retrospective study

**DOI:** 10.1186/s12893-026-04027-3

**Published:** 2026-07-07

**Authors:** Zhuang Li, Jingpeng Zhang, Wenfei Wang, Minghui Sheng, Feng Bao, Dengqun Sun

**Affiliations:** 1 Department of General Surgery, The Chinese People’s Armed Police Forces Anhui Provincial Corps Hospital, Hefei, 230041 China; 2 The First Affiliated Hospital of Anhui University of Chinese Medicine, Hefei, 230031 China

**Keywords:** Wireless intelligent endoscopic system, Gallstones, Laparoscopic cholecystectomy, Ergonomics, ERAS

## Abstract

**Objective:**

To evaluate the clinical application value of a novel wireless intelligent endoscopic system in laparoscopic cholecystectomy.

**Methods:**

A total of 86 patients undergoing cholecystectomy between September and October 2023 were enrolled and randomly allocated to the observation group (wireless group, *n* = 43) and the control group (traditional wired group, *n* = 43). We comparatively analyzed technical performance, surgical safety (Critical View of Safety (CVS) achievement rate, complications), operator fatigue (Piper Fatigue Scale), perioperative physiological indicators (liver function, stress hormones, C-reactive protein(CRP)), pain scores (Short-Form McGill Pain Questionnaire(SF-MPQ)), and patient-reported outcomes (quality of recovery (QoR-15), quality of life (GIQLI), and ERAS compliance).

**Results:**

All 86 procedures were successfully completed without conversion to open surgery or severe bile duct injury. The wireless system showed no statistical difference in image transmission latency compared to the wired system (*P >* 0.05), yet its equipment weight was significantly reduced. Preoperative preparation time was significantly shorter in the wireless group (*P <* 0.001). For surgical safety, the CVS achievement rate was 100% in both cohorts. Operator fatigue scores were significantly lower in the observation group. Furthermore, postoperative CRP levels and SF-MPQ pain scores were significantly lower in the observation group than in the control group (*P <* 0.01). Patients in the observation group also demonstrated a significant advantage in postoperative QoR-15 and GIQLI scores (*P <* 0.05), with ERAS compliance exhibiting a positive correlation with recovery quality.

**Conclusion:**

The wireless intelligent endoscopic system is safe and feasible for LC, offering a safety profile comparable to traditional wired systems. By improving surgical ergonomics and attenuating postoperative systemic inflammation and pain, the system is positively associated with patients’ early quality of recovery, effectively facilitating the implementation of the ERAS paradigm.

## Introduction

 Gallstone disease not only manifests with debilitating symptoms such as biliary colic and cholecystitis but can also progress to severe complications, including choledocholithiasis, biliary obstruction, and gallstone pancreatitis [1, 2]. These conditions significantly impair patients’ physical health and overall quality of life. Currently, laparoscopic cholecystectomy (LC) remains the gold standard and most widely utilized minimally invasive surgical technique for managing benign gallbladder diseases [3]. Driven by rapid advancements in optical, electronic, and computational technologies, minimally invasive options have further diversified to include single-incision laparoscopic surgery and natural orifice transluminal endoscopic surgery [4]. Concurrently, laparoscopic equipment itself is undergoing a paradigm shift from traditional modular configurations to intelligent wireless systems [5]. Despite these broader surgical advancements, traditional wired endoscopic systems still present inherent spatial and ergonomic limitations. The clutter of multiple interconnected cables often twists and tangles within the sterile field, thereby restricting the surgeon’s range of motion, dispersing focus, and exacerbating operative fatigue. Moreover, the frequent traction or accidental dropping of these cables can damage sensitive equipment components, leading to rapid wear and high maintenance cost [6, 7]. To overcome these physical constraints, the novel wireless endoscopic system emerges as a promising alternative. Its cable-free design inherently mitigates the risk of cross-infection, while wireless signal transmission substantially enhances surgical flexibility and operational efficiency. Additionally, the miniaturized and lightweight footprint of the device renders it highly adaptable for emergency medical interventions and diverse clinical settings [8, 9].

To overcome these physical constraints, our center has pioneered the clinical application of a novel wireless endoscopic system. Operating on a built-in 5G network, the wireless camera effectively and seamlessly transmits high-definition signals to secondary monitors with ultra-low latency. By eliminating cable clutter, this system aims to unburden the surgical field, optimize the surgeon’s maneuverability, and significantly improve intraoperative ergonomics. We hypothesize that these technical and ergonomic advantages will subsequently minimize surgical micro-trauma and perfectly synergize with the ERAS pathway.

Therefore, this study aims to systematically evaluate the clinical feasibility, safety, and ERAS optimizing potential of this novel wireless system compared to a traditional wired endoscope in a cohort of 86 patients undergoing LC.

## Methods

### Clinical data

From September 2023 to October 2023, 1038 patients underwent cholecystectomy in the Department of General Surgery at the Anhui Provincial General Hospital of the Chinese People’s Armed Police Force. Inclusion criteria: (1) Obtaining informed consent from patients and their families and voluntarily participating in the study; (2) All surgeries were performed by the same surgical team; (3) Age > 18 years old; (4) Preoperative imaging examination confirmed gallstones through liver, gallbladder, pancreas, and spleen color ultrasound or upper abdominal CT. Exclusion criteria: (1) Preoperative assessment suggests the presence of intrahepatic bile duct stones, malignant tumors or malignant transformation of the bile duct; (2) Combining severe cardiovascular disease and liver and kidney dysfunction; (3) Having mental disorders and poor compliance; (4) Individuals with surgical contraindications; (5) Early discharge and lack of postoperative clinical observation. After inclusion in the exclusion criteria, 86 patients with gallstones who underwent cholecystectomy in our hospital will be the study subjects. This study was approved by the Ethics Committee of the Anhui Provincial Hospital of the Chinese People’s Armed Police Force (No: WJYY-2023AH-04).

### Sample size calculation

Sample size calculation was performed based on the primary endpoint of postoperative CRP level. According to our preliminary clinical data, the expected postoperative CRP was 41.90 mg/L (SD = 8.20 mg/L) in conventional wired laparoscopy group and 36.80 mg/L in wireless group, with an intergroup difference of 5.10 mg/L. A two-sided α of 0.05 and statistical power of 0.80 were set for sample size estimation using the formula for two independent sample means. The calculated minimal sample size per group was 39 patients. Considering an anticipated dropout rate of 10%, 43 subjects were enrolled in each group, with a total sample size of 86 patients finally included in this prospective randomized trial. Bile duct injury (BDI) was defined as a secondary safety endpoint rather than primary outcome, because BDI is a rare adverse event with extremely low incidence in LC, which cannot be used for sample size calculation under current study design.

### Randomization and allocation concealment

All enrolled patients were randomly assigned to the observation group or control group at a 1:1 ratio using a computer-generated random number table. Random sequence was created by an independent statistician who was not involved in patient enrollment, surgical operation or outcome assessment. The grouping information was sealed in sequentially numbered opaque envelopes to implement allocation concealment. When a subject satisfied all inclusion criteria, the attending researcher opened the corresponding envelope sequentially to confirm grouping and complete enrollment, avoiding selection bias. Blinding of surgeons was not feasible due to the prominent visual differences between two endoscopic systems.

### Connection of Wireless Intelligent Endoscope

The control group was equipped with the German STORZ TC200 full-high-definition wired laparoscopic camera system. The wireless intelligent endoscope used in this study is authorized to be produced by Hefei Deming Electronics Co., Ltd. It weighs about 120 g, comes with a built-in light source system, built-in battery, low-voltage DC power supply, solves the hidden danger of AC power, and can be sterilized by low-temperature plasma. The disinfected electric cutting mirror and wireless intelligent endoscope can be directly connected for use.

### Surgical methods

**Fig. 1:**
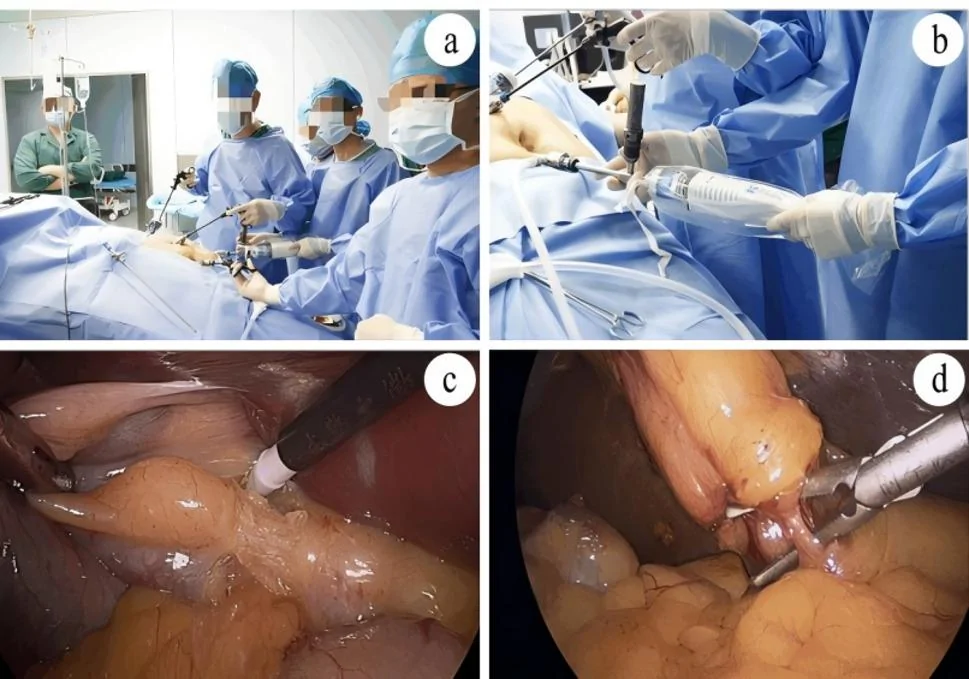
Application of wireless endoscopy in laparoscopic cholecystectomy. **a.** Using a wireless endoscope system for LC **b.** The wireless endoscope passes through the surgical area, freeing itself from the constraints of light source and camera lines, making the operation more flexible **c.** Dissecting the gallbladder triangle **d.** Freeing the cystic duct

Laparoscopic cholecystectomy was performed using both wireless and traditional wired endoscopic systems, and the surgery was performed by the same surgical team (Fig. 1). Both groups used a standard 30 degrees laparoscope with a 320 mm Length of laparoscope. Laparoscopic cholecystectomy was performed under general anesthesia. The patient was placed in a supine position and CO_2_ artificial pneumoperitoneum was established. Three port technique was used to enter the abdomen. During the operation, no exudate was found in the abdominal cavity, and no obvious abnormalities were observed in the liver, stomach, and duodenum. The gallbladder showed chronic inflammatory changes, with thick walls and clear calot triangle anatomy. The extrahepatic bile duct was also clearly displayed. The gallbladder was removed using the anterograde method, and the gallbladder duct and gallbladder artery were ligated and clamped. The gallbladder bed was electrocoagulated to stop bleeding, and after flushing the abdominal cavity without any abnormalities, the gallbladder was removed and all holes were closed. Satisfied with anesthesia, smooth operation, and safe return of the patient to the ward after surgery.

### Perioperative management

(1) All patients underwent routine preoperative evaluations to rule out surgical contraindications. In accordance with the ERAS Society guidelines [10]. (2) Postoperatively, intravenous fluid administration was restricted to less than 2000 mL within the first 48 h for both groups. An early liquid diet was initiated to maintain energy levels, and advanced patient-controlled analgesia pumps were provided for optimal pain management. Upon regaining consciousness, patients were assisted by the medical staff into a lateral decubitus position and turned regularly. In-bed exercises commenced at 6 h postoperatively, followed by out-of-bed mobilization at 12 h. Physical activity was progressively increased as tolerated to promote early flatus and gastrointestinal recovery. (3) Potential postoperative complications occurring after surgery were systematically recorded. These included gastrointestinal adverse reactions, acute pancreatitis, acute cholangitis, wound hemorrhage, surgical site infection, and bile leakage.

### Observation indicators

(1) General Demographic and Clinical Characteristics: Data on age, gender, perioperative biochemical indicators, and length of hospital stay were collected for both patient groups. (2) Subjective and Objective Performance of the Endoscopic Systems: ①Surgeon Subjective Evaluation: Thirty specialist surgeons with over 5 years of laparoscopic experience were invited to independently evaluate the operating experience of both systems. A crossover design was employed: operators first performed basic laparoscopic tasks using the wireless ultra-high-definition endoscope, followed by the same tasks using the traditional domestic wired high-definition endoscope. For the control group, the sequence was reversed. The basic tasks included a bean-dropping task, a paper-cutting task, and a suturing and knot-tying task, with each participant required to complete each task three times. Upon completion, the operators evaluated both systems based on image clarity (scored 1–5), handling ergonomics (scored 1–5), and perceived image latency. Higher scores indicate a superior operating experience. ②Objective Technical Indicators: Referencing established technical evaluation standards for minimally invasive video transmission and wireless medical devices [11], the actual image transmission latency, frame rate stability, and frequency of wireless signal interruptions for both systems were synchronously recorded and extracted using background systems and external monitoring equipment. (3) Intraoperative Outcomes and CVS Assessment: Data regarding the stone clearance rate, operative time, and the endoscopic surgeon’s Piper Fatigue Scale score were collected [12]. To objectively assess surgical safety, a blinded video evaluation of the CVS achievement was conducted. Two independent senior surgeons, not otherwise involved in the study, retrospectively reviewed and scored de-identified surgical videos in a double-blinded manner. In the event of a discrepancy between their assessments, a third senior expert intervened as an arbitrator to reach a final consensus.(4) Pain Assessment: Postoperative pain was evaluated using the SF-MPQ at preoperative baseline and postoperative day 2. The SF-MPQ was employed to comprehensively assess the severity of pain in both groups across three distinct dimensions: the Pain Rating Index (maximum 45 points), the Visual Analog Scale score (maximum 10 points), and the Present Pain Intensity (maximum 5 points). The maximum total score is 60 points, with higher scores indicating a more severe pain response. (5) Patient-Reported Outcomes (PROs): The QoR-15 questionnaire was administered on preoperative day and postoperative day 2 to evaluate the quality of recovery [13]. This scale encompasses 5 dimensions (pain, social support, emotional state, physical comfort, and physical independence), with total scores ranging from 0 to 150; higher scores indicate a better quality of recovery. Additionally, the GIQLI was utilized to comprehensively evaluate the patients’ quality of life [14]. (6) ERAS Protocol Compliance Evaluation: Based on the ERAS Society guidelines and tailored to our institutional clinical pathway for laparoscopic cholecystectomy, 12 core ERAS intervention indicators were established. These encompass preoperative education, fasting and fluid restriction management, multimodal preemptive analgesia, intraoperative goal-directed fluid therapy, hypothermia prevention, early tube removal timing, early oral feeding, and early mobilization. Independent researchers retrospectively reviewed electronic medical records to verify the execution of each item, calculating a comprehensive ERAS compliance rate for each patient.

### Indicator testing

Fasting venous blood (5 mL) was collected from all patients at preoperative baseline and 7:00 a.m. on postoperative day 2. Use the AU2700 fully automatic biochemical analyzer produced by Beckman Coulter to detect blood biochemical indicators; The Hangzhou Lianke Biological Enzyme Linked Immunosorbent Assay (ELISA) method was used to detect serum interleukin-6 (IL-6), C-reactive protein (CRP), tumor necrosis factor-α (TNF-α), and cortisol (Cor); Enzyme linked immunosorbent assay (ELISA) was used to detect serum adrenaline (E) and noradrenaline (NE) from Biogradetech in the United States.

### Statistical processing

Statistical analysis of the data was conducted using the R3.6.1 software package, and a fitted curve was drawn to represent the relationship between the compliance rate of ERAS measures and the postoperative recovery quality. Use SPSS 26.0 software to perform statistical analysis on the data. The quantitative data before and after surgery were compared using paired t-test, while the comparison between two groups was conducted using two independent sample t-test; The count data is expressed in absolute numbers and rates, and the comparison of rates between groups is performed using a chi square test. *P <* 0.05 is considered statistically significant.

## Result

### Technical performance and operator evaluation of the endoscopic systems

To address the standardized technical evaluation dimensions, we first assessed the objective engineering parameters and inherent specifications of the two systems. The Observation group demonstrated a significant advantage in physical ergonomics, with a total machine weight of only 450 g compared to 5.1 kg in the Control group. Notably, the wireless system utilized a 1/2-inch CMOS image sensor with full HD optical zoom (14–32 mm) and maintained a signal-to-noise ratio of ≥ 27 dB (Fig. 1). Crucially, objective monitoring of the image transmission revealed that the actual delay time for the wireless system was 65.71 ± 7.35 ms, which showed no statistically significant difference when compared to the 63.82 ± 8.94 ms of the wired system (*P >* 0.05). Regarding the subjective clinical experience evaluated by the operators, the wireless endoscope received significantly higher scores for image clarity (*P <* 0.05) and handling ergonomics (*P <* 0.01). Furthermore, in terms of subjective perceived latency during surgical manipulation, only 3 instances were reported in the observation group and 1 in the control group. Consistent with the objective delay measurements, this subjective perceived latency showed no statistically significant difference between the two systems (*P* > 0.05) (Table 1).

**Table 1:** Evaluation and comparison of two endoscopic systems

Variable	Observation(*n* = 43)	Control(*n* = 43)	t/x^2^	*P*
Clarity	4.75 ± 0.34	4.52 ± 0.51	2.46	0.016
Ergonomics	4.36 ± 1.12	3.67 ± 0.73	3.38	0.001
Latency			0.26	0.609
YES	3(6.98%)	1(2.33%)		
NO	40(93.02%)	42(97.67%)		
Delay(ms)	65.71 ± 7.35	63.82 ± 8.94	1.07	0.290
Whole machine weight	450 g	5.1 kg	-	-
Optical zoom	Full HD Zoom Range14～32 mm	Fixed focus 28 S	-	-
Image sensor	1/2-inch CMOS	1/3-inch CMOS	-	-
Signal to noise ratio	≥ 27dB	≧ 70dB	-	-
Image delay time	<100ms	<100ms	-	-
Screen resolution	1920*1080P	1920*1080P	-	-

### Perioperative clinical outcomes and surgical safety assessment

As detailed in Table 2, the baseline demographics and clinical characteristics, including gender, age, stone diameter, and history of chronic diseases, were comparable between the Observation and Control groups (*P >* 0.05). Interestingly, while the Observation group had a lower proportion of patients with ≥ 3 stones (*P <* 0.05), the surgical success rate was 100% in both groups. Notably, the preoperative preparation time was significantly shorter in the Observation group utilizing the wireless system compared to the Control group ( *P <* 0.001). Metrics such as intraoperative bleeding volume, extraction time of the abdominal drainage tube, length of hospital stay and time to first out-of-bed mobilization(*P >* 0.05). Postoperative patientSF-MPQ scores showed statistically significant differences between the two systems( *P <* 0.01). the endoscopic surgeon’s Piper Fatigue Scale score was significantly lower in the Observation group *(P <* 0.01), indicating that the ergonomic advantages of the wireless system effectively reduced operator fatigue during surgery. The CVS achievement rate reached 100% in both groups, indicating optimal anatomical exposure across both techniques. Furthermore, there were no cases (0%) of intraoperative bile duct injury or conversions to open surgery in either cohort.

**Table 2:** Comparison of general information of patients during the perioperative period

Variable	Observation group(*n* = 43)	Control group(*n* = 43)	t/χ^2^	*P*
Gender			0.80	0.372
Female	18(41.9%)	14(32.6%)		
Male	25(58.1%)	29(67.4%)		
Age (years)	57.1 ± 12.5	55.4 ± 14.5	0.58	0.562
Number of stones			3.84	0.050
≥ 3	20(46.5%)	29(67.4%)		
<3	23(53.5%)	14(32.6%)		
Stone diameter(cm)	1.66 ± 0.73	1.62 ± 0.67	0.26	0.792
Accompanied by a history of chronic diseases	17(39.5%)	21(48.8%)	0.75	0.385
Preoperative preparation time	3.37 ± 1.07	4.63 ± 1.05	5.51	0.000
Intraoperative bleeding volume(ml)	20.21 ± 4.45	20.45 ± 4.53	0.25	0.805
First time getting out of bed(h)	19.26 ± 4.82	18.02 ± 5.28	1.14	0.259
Extraction time of abdominal drainage tube(d)	3.26 ± 0.79	2.91 ± 0.84	1.99	0.051
Surgical success rate	43(100%)	43(100%)	-	-
Length of hospital stay(d)	6.14 ± 1.15	6.49 ± 1.70	1.12	0.267
SF-MPQ	16.26 ± 3.27	18.32 ± 3.45	2.84	0.006
Mirror doctor PFS score	4.67 ± 1.60	5.60 ± 1.43	2.84	0.006
CVS achievement rate	43(100%)	43(100%)	-	-
Bile duct injury	0	0	-	-
Conversion to open surgery	0	0	-	-

### Comparison of perioperative hepatic, stress and inflammatory profiles

Table 3 presents the comparison of perioperative liver function, stress hormones, and inflammatory markers. Before treatment, both groups exhibited elevated liver function indicators (ALT, TBil, AST, GGT) with no significant differences between them (*P >* 0.05). By postoperative day 2, these parameters had significantly decreased in both groups, confirming the efficacy of both minimally invasive techniques. No statistical differences were observed in postoperative liver function between the Observation and Control groups (all *P >* 0.05). Similarly, the systemic stress indicators (Cor, NE, and E) showed no statistically significant differences between the two groups either before or two days after treatment (*P >* 0.05), indicating comparable physiological stress responses. For inflammatory markers, baseline levels of TNF-α, IL-6, and CRP were comparable (*P >* 0.05). While postoperative TNF-α and IL-6 levels showed no significant differences between the two cohorts, the postoperative CRP level in the Observation group was significantly lower than that in the Control group (*P <* 0.01). This significant decrease highlights the potential of the wireless system to further reduce surgical micro-trauma and systemic inflammation.

**Table 3:** Perioperative liver function, stress and inflammatory markers

Variable	Observation group(*n* = 43)	Control group(*n* = 43)	t/χ^2^	*P*
ALT(U/L)				
Preoperative	57.92 ± 25.44	54.83 ± 24.28	0.58	0.566
Postoperative	40.87 ± 22.36	39.36 ± 23.0	0.31	0.758
TBil(µmol/L)				
Preoperative	21.32 ± 9.86	22.30 ± 10.35	0.45	0.654
Postoperative	15.41 ± 4.96	16.02 ± 4.86	0.58	0.566
AST(U/L)				
Preoperative	40.27 ± 12.28	41.26 ± 11.10	0.39	0.696
Postoperative	34.49 ± 10.93	32.47 ± 10.53	0.87	0.385
GGT(U/L)				
Preoperative	258.10 ± 92.90	236.98 ± 94.39	1.05	0.299
Postoperative	189.95 ± 108.54	182.95 ± 98.97	0.31	0.755
E(nmol/L)				
Preoperative	1.23 ± 0.52	1.40 ± 0.41	1.68	0.096
After treatment	1.63 ± 0.74	1.57 ± 0.39	0.47	0.639
NE(nmol/L)				
Preoperative	1.96 ± 0.36	2.12 ± 0.87	1.11	0.268
Postoperative	1.75 ± 0.41	1.78 ± 0.49	0.31	0.759
Cor(mmol/L)				
Preoperative	201.25 ± 75.32	198.16 ± 72.98	0.19	0.847
Postoperative	161.16 ± 89.56	155.07 ± 77.56	0.34	0.737
TNF-α(ng/L)				
Preoperative	25.73 ± 8.39	26.09 ± 9.12	0.19	0.849
Postoperative	22.60 ± 5.92	21.74 ± 7.17	0.61	0.546
IL-6(ng/L)				
Preoperative	43.89 ± 6.00	43.76 ± 5.06	0.11	0.914
Postoperative	41.26 ± 5.35	39.84 ± 10.22	0.81	0.422
CRP(mg/L)				
Preoperative	42.34 ± 10.34	45.27 ± 8.92	1.41	0.163
Postoperative	36.75 ± 7.60	41.86 ± 8.15	3.01	0.004

### ERAS protocol compliance and patient-reported outcomes

**Fig. 2:**
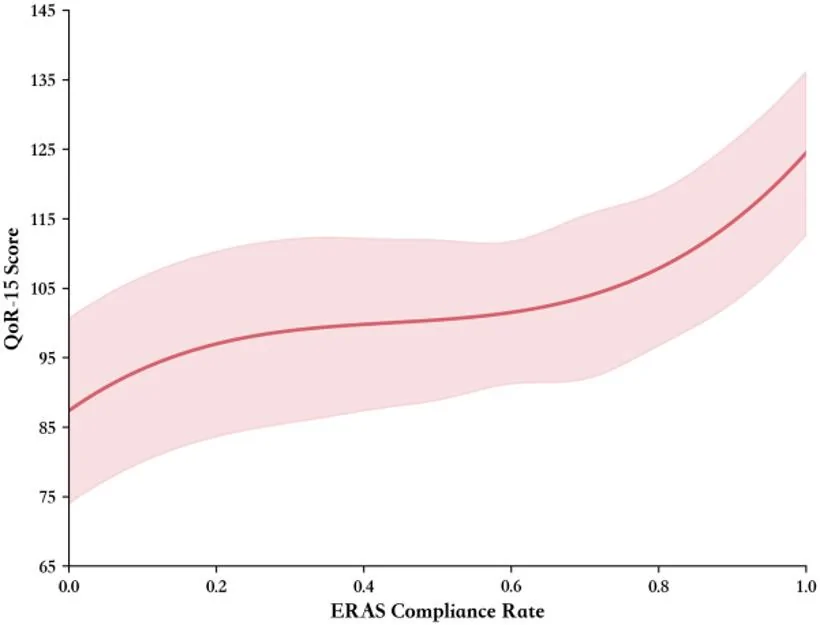
The relationship between ERAS protocol compliance rate and postoperative QoR-15 score

At the preoperative baseline, there were no statistically significant differences between the Observation and Control groups in either the QoR-15 scores (*P >* 0.05) or the GIQLI scores (*P >* 0.05), ensuring excellent comparability between the two cohorts. Postoperatively, patients in the Observation group demonstrated a significantly higher QoR-15 score compared to those in the Control group (*P <* 0.01). This crucial PROs metric indicates a superior early quality of recovery. Furthermore, the postoperative GIQLI score was also significantly elevated in the Observation group (*P <* 0.05) (Table 4). They confirm effectively translate into enhanced subjective postoperative comfort and an accelerated return to a high quality of life.Crucially, to elucidate the clinical impact of adhering to the ERAS pathway, we analyzed the correlation between the ERAS compliance rate and the primary PROs metric. As illustrated in Fig. 2, the restricted cubic spline curve demonstrates a robust positive association between the two variables. Notably, as the ERAS compliance rate increases, the postoperative QoR-15 score exhibits a substantial and continuous upward trend. This correlation elegantly confirms that optimizing ERAS compliance—driven by the technical advantages of the wireless endoscopic system—directly and effectively enhances subjective patient recovery.

**Table 4:** Comparison of perioperative patient-reported outcomes between the two groups

Variable	Observation group(*n* = 43)	Control group(*n* = 43)	t/x2	*P*
QoR-15				
Preoperative	115.59 ± 7.78	113.13 ± 5.19	1.72	0.088
Postoperative	134.19 ± 4.08	131.26 ± 5.65	2.76	0.007
GIQLI				
Preoperative	74.09 ± 4.24	75.68 ± 4.52	1.68	0.096
Postoperative	105.64 ± 7.43	102.11 ± 4.78	2.62	0.010

### Comparison of the incidence of complications between two groups of patients

Within one month after surgery, there were no complications, including gastrointestinal reactions, acute pancreatitis, acute cholangitis, wound bleeding, wound infection, bile leakage.

### Awareness and acceptability

According to the questionnaires from the 43 participants Observation group, only 4 participants (9.3%) were familiar with wireless endoscope system and more than half of the participants did not know much about wireless endoscope system before this trial. After takingwireless endoscope system examinations, all participants found this novel method acceptable, with 78% of the participants considering wireless endoscope system very necessary and the other 22% considering it necessary.

## Discussions

Acute calculous cholecystitis requires prompt surgical intervention to relieve biliary obstruction and infection [15]. While effective, traditional open cholecystectomy entails significant trauma, elevated infection risks, and prolonged recovery [16]. Consequently, LC has become the gold standard for benign gallbladder diseases due to its minimal invasiveness and rapid recovery profile [17]. However, traditional wired endoscopic systems impose inherent physical constraints. Cable clutter and tethered mobility restrict the surgeon’s range of motion, compromising operative efficiency and ergonomics. These limitations present a critical bottleneck in the advancement of precision minimally invasive surgery [18, 19]. To address the paradigm shift toward untethered surgical platforms, this study evaluated the clinical utility of a novel wireless intelligent endoscopic system in LC. Moving beyond standard perioperative metrics, we applied a multidimensional framework encompassing technical performance, surgical ergonomics, and patient-centered outcomes. Our findings demonstrate that the wireless system ensures reliable signal transmission and surgical safety while significantly reducing operator fatigue. Notably, this ergonomic optimization directly translates into attenuated systemic inflammation, higher compliance with ERAS protocols, and improved early postoperative recovery. These results highlight the potential of wireless technology to foster a more precise, untethered, and holistic surgical recovery paradigm.

The rapid integration of 5G and 4 K imaging technologies has facilitated the adoption of wireless intelligent devices in surgical practice [20, 21]. Recent urological studies have demonstrated that wireless ultra-high-definition laparoscopic systems eliminate signal latency and deliver superior image clarity compared to traditional wired counterparts, yielding favorable clinical outcomes across various minimally invasive procedures [7, 22, 23]. Building upon this foundation, we systematically applied this technological paradigm to LC. Our results demonstrated a 100% CVS achievement rate and a 0% incidence of bile duct injury in the wireless group, confirming that its safety profile is completely comparable to the conventional wired gold standard. These robust clinical data not only corroborate prior expectations regarding the safety of wireless technology but also effectively dispel concerns about its routine clinical application. The core value distinguishing our study from previous literature is the demonstration that this operational optimization extends beyond a mere improvement in surgeon experience. Instead, it bridges the traditional disconnect between surgical ergonomics and patient recovery, establishing a functional linkage where optimized operative performance directly minimizes patient trauma.

While perioperative liver function and stress hormones remained comparable between the groups, the significantly lower postoperative CRP levels in the Observation group underscore the system’s clinical advantage in minimizing surgical trauma. The mechanisms driving this attenuated inflammatory response can be elucidated through both tissue handling and biomechanical perspectives. The ergonomic freedom afforded by the wireless system enables more meticulous and gentle tissue dissection, mitigating excessive traction on the gallbladder bed and collateral thermal damage. Crucially, this aligns with the concept of micro-ergonomic optimization proposed by our research team [24]. Traditional wired systems exert continuous pressure and mechanical traction on abdominal wall trocars due to the heavy weight of the cables. By eliminating this mechanical force, the wireless system prevents the subsequent localized sterile inflammatory cascade, which manifests systemically as reduced CRP levels—and physically mitigates trocar-site incisional pain [25]. This synergistic reduction in inflammation and pain provides a vital biological substrate for optimizing the ERAS model, advancing minimally invasive surgery toward a new dimension of enhanced patient comfort.

This study acknowledges several limitations. The limited sample size and single-center design may restrict the generalizability of the findings, necessitating further validation through multicenter, large-scale prospective cohort studies; Long-term follow-up to assess delayed postoperative complications is currently lacking. Looking ahead, the integration of artificial intelligence and robotic assistance with wireless endoscopic platforms holds immense potential for achieving intelligent surgical navigation and precise operative control. This technological synergy will propel minimally invasive surgery from an era of precision into a new paradigm of intelligent interventions.

## Conclusion

The wireless intelligent endoscopic system is safe and feasible for LC, demonstrating surgical success, bile duct safety, and physiological stability comparable to the wired gold standard. Its lightweight, untethered design improves surgical ergonomics, significantly reducing preoperative preparation time and operator fatigue. Importantly, the application of this system is positively associated with attenuated systemic inflammation, reduced incisional pain, and improved early recovery and overall quality of life. By eliminating the physical constraints of traditional cabling, the wireless system facilitates adherence to ERAS protocols. Furthermore, the positive correlation observed between ERAS compliance and recovery quality suggests that this untethered technology may effectively support a precise and patient-centered surgical paradigm.

## Data Availability

The datasets used and/or analyzed during the current study are available from the corresponding author on reasonable request. The data are not publicly available due to ethical and privacy restrictions.

## Abbreviations

ALT: Alanine aminotransferase
TBil: Total bilirubin
AST: Aspartate aminotransferase
GGT: Glutamyl transpeptidase
CRP: C-reactive protein
Cor: Cortisol
NE: Norepinephrine
E: Adrenaline
PFS: Piper Fatigue Scale
ELISA: Enzyme Linked Immunosorbent Assay
LC: Laparoscopic Cholecystectomy
PROs: Patient-Reported Outcomes
SF-MPQ: Short-Form McGill Pain Questionnaire

## References

[1] Ji H, Hou YF, Cheng XJ, Zhu F, Wan C, Fang L. Association of Laparoscopic Methods and Clinical Outcomes of Cholecystolithiasis Plus Choledocholithiasis: A Cohort Study. Turkish J Gastroenterol. 2023;34(1):35–42. 10.5152/tjg.2022.22110 PMC9985062 36445052

[2] McDermott J, Kao LS, Keeley JA, Nahmias J, de Virgilio C. Management of Gallstone Pancreatitis. Jama Surg. 2024;159(7):818–25. 10.1001/jamasurg.2023.8111 38691369

[3] Yuan Y, Shi G, Chen H, Wang M, Liu H, Zhang X, Wang B, Zhang G, Sun L. Effects of preoperative oral enzyme-hydrolyzed rice flour solution on gastric emptying and insulin resistance in patients undergoing laparoscopic cholecystectomy: a prospective randomized controlled trial. BMC Anesthesiol. 2023;23(1):52. 10.1186/s12871-023-02012-4 PMC9923920 36782111

[4] Nam C, Lee JS, Kim JS, Lee TY, Yoon YC. Evolution of minimally invasive cholecystectomy: a narrative review. BMC Surg. 2024;24(1):378. 10.1186/s12893-024-02659-x PMC11606199 39609785

[5] Kim HKJ, Abraham A, DeCicco J, Haas AJ, Pollard R, El-Hayek K. First reported human use of wireless laparoscopic system: is it ready for prime time? Surg Endoscopy Other Interventional Techniques. 2024;38(11):6918–22. 10.1007/s00464-024-11286-9 PMC11525237 39327296

[6] Noordmans HJ, de Braak M, Brakel MJ, Onur AR, De Geus J, Jaspers JEN. Multi-Center Evaluation of Optical Degradation Patterns in Rigid Endoscopes: A Data-Driven Approach. Med Devices-Evidence Res. 2025;18:507–18. 10.2147/MDER.S550187 PMC12517457 41089934

[7] Niu D, Xu Q, Xu H, Yin S, Hao Z, Shi H, Zhou J, Tai S, Zou Z, Yang C, et al. Fabrication and application of a wireless high-definition endoscopic system in urological surgeries. BJU Int. 2023;131(2):183–9. 10.1111/bju.15718 PMC10078773 35199469

[8] Lacy AM, Bravo R, Otero-Pineiro AM, Pena R, De lacy FB, Menchaca R, Balibrea JM. 5G-assisted telementored surgery. Br J Surg. 2019;106(12):1576–9. 10.1002/bjs.11364 31483054

[9] Maimon NB, Bez M, Drobot D, Molcho L, Intrator N, Kakiashvilli E, Bickel A. Continuous Monitoring of Mental Load During Virtual Simulator Training for Laparoscopic Surgery Reflects Laparoscopic Dexterity: A Comparative Study Using a Novel Wireless Device. Front NeuroSci. 2022;15:694010. 10.3389/fnins.2021.694010 PMC8811150 35126032

[10] Joliat G-R, Kobayashi K, Hasegawa K, Thomson J-E, Padbury R, Scott M, Brustia R, Scatton O, Cao HST, Vauthey J-N, et al. Guidelines for Perioperative Care for Liver Surgery: Enhanced Recovery After Surgery (ERAS) Society Recommendations 2022. World J Surg. 2023;47(1):11–34. 10.1007/s00268-022-06732-5 PMC9726826 36310325

[11] Chen Y, Zhang T, Jiang X, He C, Hu J-P, Liao Z, Li Z-S. 5G-based remote magnetically controlled capsule endoscopy for examination of the stomach and small bowel. J Gastroenterol Hepatol. 2023;38:22–22. 10.1002/ueg2.12339 PMC9892422 36416805

[12] Mustian KM, Alfano CM, Heckler C, Kleckner AS, Kleckner IR, Leach CR, Mohr D, Palesh OG, Peppone LJ, Piper BF, et al. Comparison of Pharmaceutical, Psychological, and Exercise Treatments for Cancer-Related Fatigue A Meta-analysis. Jama Oncol. 2017;3(7):961–8. 10.1001/jamaoncol.2016.6914 PMC5557289 28253393

[13] Nyundo M, Kayondo K, Gasakure M, Twagirumukiza JD, Gashegu J, Detry O. Implementation and outcomes of an enhanced recovery after surgery pathway for laparoscopic cholecystectomy in East and Central Africa: A prospective non-randomized controlled trial in Rwanda’s Tertiary Teaching Hospital. World J Surg. 2025;49(3):605–14. 10.1002/wjs.12371 39390308

[14] Liao J-H, Li J-S, Wang T-L, Liu W-S. Laparoscopic cholecystectomy plus common bile duct exploration for extrahepatic bile duct stones and postoperative recurrence-associated risk factors. World J Gastrointest Surg. 2024;16(11):3511–9. 10.4240/wjgs.v16.i11.3511 PMC11622081 39649185

[15] Yan Y, Hua Y, Yuan W, Zhu X, Du Y, Zhu S, Wang B. Utility of Tokyo Guidelines 2018 in early laparoscopic cholecystectomy for mild and moderate acute calculus cholecystitis: A retrospective cohort study. Front Surg. 2023;9:1022258. 10.3389/fsurg.2022.1022258 PMC9885794 36726949

[16] Li Z, Qiao Z, Wang W, Sun Y, Sun D, Sheng M. Breaking age barriers: the efficacy of ERCP in advanced age patients. BMC Surg. 2025;25(1):342. 10.1186/s12893-025-03083-5 PMC12326853 40770712

[17] Pesce A, Ramirez-Giraldo C. Which is the best timing for performing a cholecystectomy after percutaneous cholecystostomy? Surg Endoscopy Other Interventional Techniques. 2025;39(1):14–5. 10.1007/s00464-024-11432-3 39658671

[18] Li N, Liu H, Abdolmalaki RY, Mancini GJ, Tan J. s-CAM: An Untethered Insertable Laparoscopic Surgical Camera Robot with Non-Contact Actuation. Sensors 2022; 22(9). 10.3390/s22093405 PMC9100798 35591095

[19] Balafoutas D, Joukhadar R, Vlahos N. Optimising ergonomics in minimally invasive gynaecological surgery: a comprehensive review and practice recommendations. Facts Views Vis Obgyn. 2025;17(2):180–91. 10.52054/FVVO.2025.12 PMC12233109 40552736

[20] Rayyan MM, Hussien ANM, Sayed NM, Abdallah R, Osman E, El Saad NA, Ramadan S. Comparison of four cordless gingival displacement systems: A clinical study. J Prosthet Dent. 2019;121(2):265–70. 10.1016/j.prosdent.2018.05.010 30722986

[21] Singla V, Bhattacharjee HK, Gupta E, Singh D, Mishra AK, Kumar D. Performance of three-dimensional and ultra-high-definition (4K) technology in laparoscopic surgery: A systematic review and meta-analysis. J Minim Access Surg. 2022;18(2):167–75. 10.4103/jmas.jmas_122_21 PMC8973492 35313429

[22] Intelligent Equipment Technology Bramch of China Medical, Equipment D. Andrology, Sexual Medicine Branch of the Chinese Medical D, Association: Clinical practice standard and specifications for wireless intelligent ultra high-definition endoscopic surgery in urology. Zhonghua yi xue za zhi. 2023;103(14):1027–34. 10.3760/cma.j.cn112137-20221212-02624 37032152

[23] Kim J-H, Nam S-J. Capsule Endoscopy for Gastric Evaluation. Diagnostics. 2021;11(10):1792. 10.3390/diagnostics11101792 PMC8534557 34679491

[24] Liu S, Li Y-y, Li D, Wang F-Y, Fan L-J, Zhou L. -x: Advances in objective assessment of ergonomics in endoscopic surgery: a review. Front Public Health. 2024;11:1281194. 10.3389/fpubh.2023.1281194 PMC10796503 38249363

[25] Myla K, Bou-Ayash N, Kim WC, Bugaev N, Bawazeer M. Is implementation of robotic-assisted procedures in acute care general surgery cost-effective? J Robotic Surg. 2024;18(1):223. 10.1007/s11701-024-01912-y 38801638

